# The Influence Of Intrauterine Pressure On Embryo Retention In A Catheter After Embryo Transfer

**DOI:** 10.1038/s41598-019-48077-5

**Published:** 2019-08-19

**Authors:** Małgorzata Kozikowska, Miroslaw Grusza, Grzegorz Mrugacz, Jerzy Gagan, Monika Zbucka-Krętowska, Cezary Grygoruk

**Affiliations:** 1 Center for Reproductive Medicine Bocian, Akademicka 26, Bialystok, Poland; 2 Faculty of Mechanical Engineering, Bialystok Technical University, Wiejska 45A, Bialystok, Poland; 3 Department of Reproduction and Gynecological Endocrinology, Sklodowskiej Curie 24a, Medical University of Bialystok, Bialystok, Poland

**Keywords:** Image processing, Outcomes research

## Abstract

The retention of the embryo in the transfer catheter after embryo transfer (ET) during *in vitro* fertilization is a very common phenomenon, encountered by even the most experienced operators, and embryos retained in the transfer catheter or its sleeve require a repeat transfer. The exact mechanism of embryo retention has not been explained. Therefore, the present study aimed to investigate the mechanism of embryo retention in the catheter during embryo transfer by using a transparent uterus model equipped with pressure sensors and a video recorder. The results indicate that pressure changes in the uterine cavity during ET can influence the distribution of the transferred fluid containing the embryo. Under certain conditions, the transferred fluid can flow backward in the catheter, which may lead to retention of the embryo in the catheter.

## Introduction

Embryo transfer (ET) has been recognized as a vital step that influences pregnancy rates in patients undergoing *in vitro* fertilization (IVF).^1,2^ To date, however, other stages of IVF have received more attention than ET, such as nutrition supplementation,^3,4^ ovarian stimulation,^5,6^ embryo incubation conditions,^7,8^ endometrial scratching,^9,10^ and luteal phase support.^11,12^ Undoubtedly, the safe placement of embryos in the uterine cavity is essential to obtaining an optimal pregnancy rate, but there is no guarantee that embryos will remain in the uterine cavity after the procedure. Embryos have been found in catheters, on the cervix, and on the vaginal speculum.^13,14^ Embryo retention (ER) in the catheter is a common phenomenon, encountered by even the most experienced operators. The reported incidence of retained embryos ranges from 1% to 7% in ET with cleavage-stage embryos.^13–19^ Embryos retained in the transfer catheter or its sleeve require a repeat transfer, and, according to some authors, ER in the transfer set can significantly reduce the pregnancy rate.^13,14^ The exact mechanism of ER has not been explained, but it may be assumed that the current design of catheters may favor ER in certain circumstances. On the other hand, the environment in the uterine cavity, especially pressure changes resulting from periodic smooth muscle contractions, may be important in ER.

Taking into consideration the high incidence of ER and its potential influence on treatment outcomes, it is crucial to understand the cause of the problem. Therefore, the current work studies the influence on ER of pressure changes in the uterine cavity.

## Results

ET was repeated 10 times for each studied pressure conditions, and the findings were constant independently of the variations in transfer technique.

**Figure 1:**
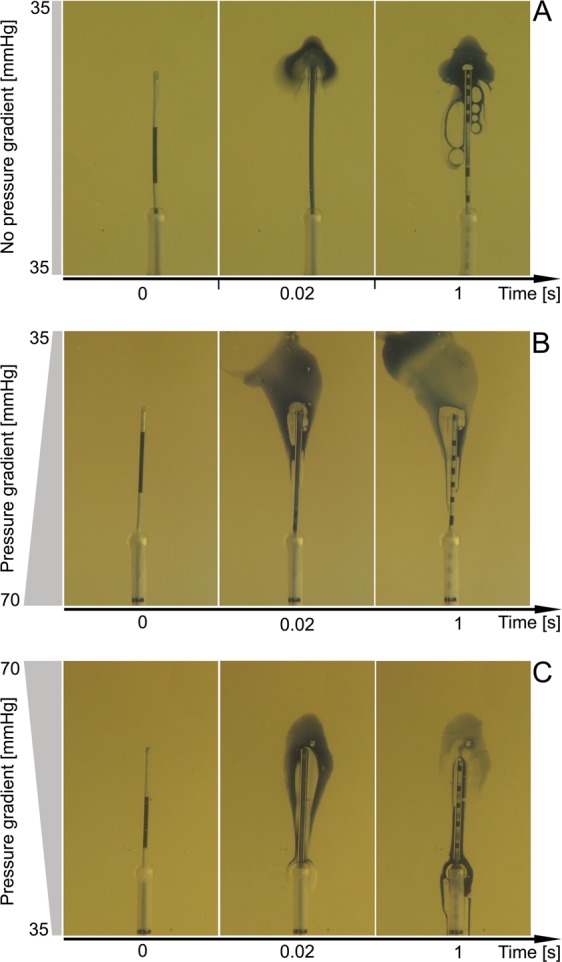
Propagation of the transferred load within the model of the uterine cavity. (**A**) no pressure-gradient force between proximal and distal parts of the model; (**B**) lower pressure in distal (35 mmHg) than proximal (70 mmHg) parts of the model; and (**C**) higher pressure in distal (70 mmHg) than proximal (35 mmHg) parts of the model.

**Figure 2:**
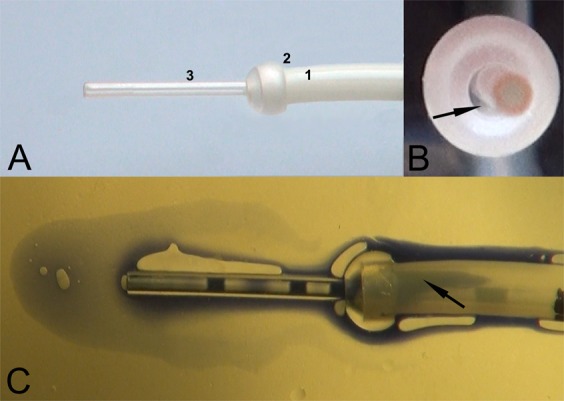
Sites of embryo retention and fluid propagation during embryo transfer. (**A**) Sites of embryo retention in descending order of occurrence: 1 - space between inner catheter and catheter sheath, 2 – surface of the catheter sheath, 3 – surface of the inner catheter; (**B**) Anterior view of the embryo transfer catheter. An arrow indicates the space between the inner catheter and catheter sheath; and (**C**) Propagation of the transferred load inside the uterine cavity model with pressure-gradient force directed toward the catheter. An arrow indicates the transferred liquid in the space between the inner catheter and catheter sheath.

When the pressure in the uterine model was set to 35 mmHg and there was no pressure gradient between the proximal and distal parts, the transferred fluid accumulated mainly around the tip of the inner catheter at 0.02 s after injection. Subsequently, at 1 s, most of the fluid being transferred remained around the catheter tip, but some of the fluid and air bubbles moved along the inner catheter toward the outer sheath, although they did not reach it (Fig. 1A). Overall, the transferred fluid reached only one of the three possible sites of ER: the surface of the inner catheter (Fig. 2A).

When the pressure was higher in the proximal (70 mmHg) than in the distal (35 mmHg) part of the uterus model and the pressure-gradient force was directed away from the catheter tip, the transferred fluid moved markedly away from the catheter tip, and only a fraction of it remained around the tip of the inner catheter at 0.02 s after injection. Subsequently, at 1 s, most of the fluid being transferred moved away from the catheter; a small portion moved along the inner catheter toward the outer sheath but did not reach it (Fig. 1B). Interestingly, the air contained in the transferred volume separated from the fluid and gathered around the catheter tip. Overall, the transferred fluid could reach only one of the three possible sites of ER: the surface of the inner catheter (Fig. 2A).

Injection of the transferred fluid when the pressure was higher in the distal (75 mmHg) than in the proximal (35 mmHg) part of the uterus model and the pressure-gradient force was directed toward the catheter predisposed the transferred liquid to move back along the catheter in the direction of the proximal part of the model (Figs 1C and 2C). Initially, the transferred load gathered around the catheter tip with a marked backward flow along the catheter and reached the outer sheath (Fig. 1C). Subsequently, at 1 s, the transferred load continued backward along the catheter sheath to reach the surface of the sheath and the space between the inner catheter and the sheath (Figs 1C and 2C). Overall, the transferred fluid could reach all three sites of ER: the surface of the inner catheter, the space between the inner catheter and the catheter sheath, and surface of the catheter sheath (Figs 1C and 2C).

## Discussion

The results of the present study indicate that pressure changes in the uterine cavity during ET can influence the distribution of the transferred fluid. In certain conditions, the transferred fluid can flow backward along the catheter, which may lead to retention of the embryo in the catheter.

The uterus is composed mostly of smooth muscles that exhibit spontaneous and rhythmic waves of contraction and relaxation.^20^ Therefore, the pressure in the uterine cavity fluctuates. A variation in pressure between distinct parts of the uterus causes a pressure-gradient force, which is always directed from the region of higher pressure to that of lower pressure. The pressure-gradient force is responsible for moving fluid in the uterus (for example, during menstruation). Taking into account that uterine contractions also occur in the early luteal phase, the pressure-gradient force can influence the distribution of the transferred load in the uterine cavity during ET.

The experimental model applied in the present study enabled us to simulate ET in changing pressure conditions. Three circumstances were considered during the study: no pressure-gradient force, a pressure-gradient force directed toward the catheter, and a pressure-gradient force directed away from catheter. According our results, a pressure-gradient force directed toward the catheter constitutes the highest risk for ER, as it predisposes the transferred load to move back along the catheter toward the surface of the catheter sheath and the space between the inner catheter and the sheath (Figs 1C and 2C). Based on our observation (unpublished data), the most common sites of ER in the catheter are the space between the inner catheter and the catheter sheath (80%), the surface of the catheter sheath (12%), and the surface of the inner catheter (8%) (Fig. 2). All three of these ER sites can be reached by the transferred liquid when the pressure-gradient force is directed toward the catheter. On the other hand, when there was no pressure-gradient force or when it was directed away from the catheter, the transferred fluid was able to reach only the least common site of ER: the surface of the inner catheter (Fig. 1A,B).

The major limitation of the study is that the model may not confirm the exact place where the embryo is retained after ET, as the experimental model evaluates only the fluid distribution. In addition, the shape of the uterine cavity is not entirely flat, and the pressure distribution in the uterine cavity may be more complex than that studied.

Furthermore, in order for an embryo to stick to the inner catheter or the outer sheath of the catheter, blood or mucous is necessary as an adhesive. The cervical tissue secrets mucous, and the outer sheath of the catheter may become encumbered with mucus or blood when it passes through the opening of the cervix. As a result, the inner catheter may become covered or clogged with mucus when it clears the outer sheath of the catheter. The mucus may then interfere with the transfer of the embryo, with the embryo possibly sticking to the mucus and not being transferred to the uterus. Neither blood nor mucous is necessary to trap the embryo in the space between the outer sheath and the inner catheter, however. Furthermore, the backward flow of fluid along the catheter during ET can be a predisposing factor for the subsequent transfer of the embryo to the cervix or the vagina upon withdrawal of the catheter.^13^

Taking into consideration the results of the present study, it would be advisable to perform ET when the uterus is relaxed, without contractions that alter the pressure in the uterine cavity. Pregnancy rates after IVF-ET decrease in a stepwise fashion with the increasing frequency of uterine contractions.^21^ In ET recipients, in addition to spontaneous contractions, the uterus is exposed to strong contractile stimuli arising from the transcervical passage of the transfer catheter and, in some cases, from using a tenaculum.^5,6^ To achieve maximal relaxation of the uterus during ET, it is advised to perform ET gently, without excessive stimulation of the cervix. Other methods to inhibit excessive uterine contractions include lowering patient stress and using a myorelaxant, acupuncture, or an oxytocin antagonist.^22,23^ Based on the results of present study, it may be assumed that the withdrawal of the catheter’s sheath before the injection of the embryo into the uterine cavity may reduce the incidence of ER. Even if that is true, however, one should keep in mind that the catheter sheath is a kind of blockade against the embryo’s moving in the direction of the cervix, so removing the sheath may increase the incidence of deposition of the embryo in the cervix.

Due to the relatively high incidence of ER, it should be routine laboratory procedure to flush the catheter with a culture medium after ET to check for the retained embryo. Furthermore, according to recent evidence, transvaginal ultrasound guidance of the ET significantly increases the percentage of pregnancies per transfer.^24,25^ Soft catheters seem to improve outcomes compared with stiff ones, and the inclusion of hyaluronan in the ET medium increases successful implantation.^26,27^

In conclusion, the results of the present study for the first time identify uterine contractions and changing pressure conditions as a possible cause of ER. Therefore, it is reasonable to suggest transferring embryos when the uterus is idle to minimize the risk of ER. In addition, it may be prudent to redesign the ET catheter to one that either eliminates or significantly lessens the risk of ER.

## Methods

**Figure 3:**
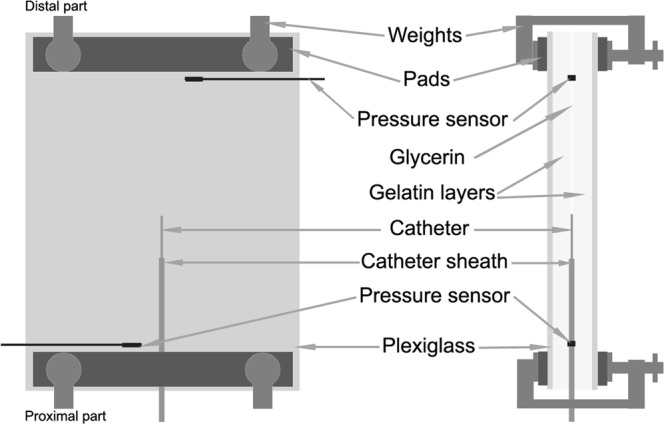
Schematic description of the experimental setup for the simulation of embryo transfer.

The experimental setup for the simulation of ET comprised a uterine cavity model, an ET catheter (Labotect GmbH, Germany), two pressure sensors and pressure transducers (Mikro-Tip® pressure transducer catheters, Millar, Houston, Texas, USA), a pressure-control unit (PCU-2000 pressure-control unit with patient isolation, Millar, Houston, Texas, USA), a data-acquisition system (PowerLab 4/30, Millar, Houston, Texas, USA), and a camcorder (Sony CX675, Japan). The uterine cavity model was composed of two plexiglass plates covered with a 5-mm layer of gelatin (50 g/250 ml, G1890 Sigma). The thin space between the opposing gelatin layers was filled with glycerin, as the uterine fluid is assumed to have similar properties.^28^ The insulin syringe (1 ml) was connected to a catheter and loaded with a combination of air and embryo culture medium (G-IVF, Vitrolife) on the basis of a routine clinical protocol previously described.^29–31^ The transferred liquid was stained with methylene blue for better visualization. The ET catheter and two pressure sensors were positioned inside the model of the uterus as presented in Fig. 3.

The pressure in the uterus model was adjusted according to experimental requirements by the use of weights (Fig. 3). The base pressure was set at 35 mmHg and the maximal pressure at 70 mmHg, which corresponds to pressure values recorded in the uterine cavity in the early luteal phase of the cycle.^32^ The mock ETs were performed at three distinct pressure settings in the model of the uterine cavity: (a) equal pressure of 35 mmHg in the proximal and distal parts of the uterus model; (b) lower pressure in the distal (35 mmHg) than in the proximal (70 mmHg) part of the uterus model; and (c) higher pressure in the distal (70 mmHg) than in the proximal (35 mmHg) part of the uterus model. The mock transfer procedures were performed by a medical doctor according to his routine clinical practice. The practitioner kept his finger on the plunger until removing the catheter from the uterus model, and the inclination of the model was not altered during the experiments. The propagation of transferred liquid in the uterine cavity model was recorded by the camcorder, and the video recordings were analyzed with video editing software (Pinnacle Studio, Corel Inc., USA). ET was repeated 10 times for each studied pressure condition. The ability of the transferred liquid to reach the ER sites was assessed, and the following ER sites were taken into consideration: the space between the inner catheter and the catheter sheath, the surface of the catheter sheath, and the surface of the inner catheter (Fig. 2A).

All procedures were carried out in accordance with relevant guidelines and regulations.

## References

[1] Mansour R, Aboulghar M (2002). Optimizing the embryo transfer technique. Human reproduction.

[2] Neithardt AB, Segars JH, Hennessy S, James AN, McKeeby JL (2005). Embryo afterloading: a refinement in embryo transfer technique that may increase clinical pregnancy. Fertility and sterility.

[3] Vitale, S. G. *et al*. How to achieve high-quality oocytes? The key role of myo-inositol and melatonin. *International journal of endocrinology* **2016** (2016). 10.1155/2016/4987436 PMC5019888 27651794

[4] Zheng Xiangqin, Lin Danmei, Zhang Yulong, Lin Yuan, Song Jianrong, Li Suyu, Sun Yan (2017). Inositol supplement improves clinical pregnancy rate in infertile women undergoing ovulation induction for ICSI or IVF-ET. Medicine.

[5] Bordewijk E, Mol F, van der Veen F, Van Wely M (2019). Required amount of rFSH, HP-hMG and HP-FSH to reach a live birth: a systematic review and meta-analysis. Human Reproduction Open.

[6] Papaleo E (2016). Clinical application of a nomogram based on age, serum FSH and AMH to select the FSH starting dose in IVF/ICSI cycles: a retrospective two-centres study. European Journal of Obstetrics & Gynecology and Reproductive Biology.

[7] Alhelou Y, Adenan NAM, Ali J (2018). Embryo culture conditions are significantly improved during uninterrupted incubation: A randomized controlled trial. Reproductive biology.

[8] Insua MF (2017). Obstetric and perinatal outcomes of pregnancies conceived with embryos cultured in a time-lapse monitoring system. Fertility and sterility.

[9] Vitagliano A (2018). Endometrial scratch injury before intrauterine insemination: is it time to re-evaluate its value? Evidence from a systematic review and meta-analysis of randomized controlled trials. Fertility and sterility.

[10] Vitagliano A (2018). Does endometrial scratching really improve intrauterine insemination outcome? Injury timing can make a huge difference. Journal of gynecology obstetrics and human reproduction.

[11] Mohammed, A. *et al*. Evaluation of progestogen supplementation for luteal phase support in fresh *in vitro* fertilization cycles. *Fertility and Sterility* (2019) in press. 10.1016/j.fertnstert.2019.04.021 31200970

[12] van der Linden, M., Buckingham, K., Farquhar, C., Kremer, J. A. & Metwally, M. Luteal phase support for assisted reproduction cycles. *Cochrane Database of Systematic Reviews* **7**, 10.1002/14651858.CD009154.pub3 (2015). 10.1002/14651858.CD009154.pub3 PMC6461197 26148507

[13] Poindexter AN (1986). Residual embryos in failed embryo transfer. Fertility and sterility.

[14] Visser DS, Fourie FL, Kruger HF (1993). Multiple attempts at embryo transfer: effect on pregnancy outcome in an *in vitro* fertilization and embryo transfer program. Journal of assisted reproduction and genetics.

[15] Lee H-C, Seifer DB, Shelden RM (2004). Impact of retained embryos on the outcome of assisted reproductive technologies. Fertility and sterility.

[16] Nabi A, Awonuga A, Birch H, Barlow S, Stewart B (1997). Multiple attempts at embryo transfer: does this affect *in-vitro* fertilization treatment outcome?. Human reproduction.

[17] Silberstein T (2005). Ultrasound-guided miduterine cavity embryo transfer is associated with a decreased incidence of retained embryos in the transfer catheter. Fertility and sterility.

[18] Tur-Kaspa I (1998). Difficult or repeated sequential embryo transfers do not adversely affect *in-vitro* fertilization pregnancy rates or outcome. Human reproduction.

[19] Vicdan K (2007). The effect of retained embryos on pregnancy outcome in an *in vitro* fertilization and embryo transfer program. European Journal of Obstetrics & Gynecology and Reproductive Biology.

[20] Kelly JV (1962). Myometrial participation in human sperm transport: a dilemma. Fertility and sterility.

[21] Fanchin R (1998). Uterine contractions at the time of embryo transfer alter pregnancy rates after *in-vitro* fertilization. Human reproduction (Oxford, England).

[22] Dieterle S, Ying G, Hatzmann W, Neuer A (2006). Effect of acupuncture on the outcome of *in vitro* fertilization and intracytoplasmic sperm injection: a randomized, prospective, controlled clinical study. Fertility and sterility.

[23] Pierzynski P, Reinheimer TM, Kuczynski W (2007). Oxytocin antagonists may improve infertility treatment. Fertility and sterility.

[24] Cozzolino M (2018). Ultrasound-guided embryo transfer: summary of the evidence and new perspectives. A systematic review and meta-analysis. Reproductive biomedicine online.

[25] Larue L (2017). Transvaginal ultrasound-guided embryo transfer in IVF. Journal of gynecology obstetrics and human reproduction.

[26] Bontekoe, S., Blake, D., Heineman, M. J., Williams, E. C. & Johnson, N. Adherence compounds in embryo transfer media for assisted reproductive technologies. *Cochrane database of systematic reviews* **7**, 10.1002/14651858.CD007421.pub2 (2010). 10.1002/14651858.CD007421.pub2 20614459

[27] Buckett WM (2006). A review and meta-analysis of prospective trials comparing different catheters used for embryo transfer. Fertility and sterility.

[28] Allen W, Tiplady C, Butler S, Mackley M (2002). Rheological characterisation of estrous uterine fluid in the mare. Theriogenology.

[29] Grygoruk C (2012). Influence of embryo transfer on embryo preimplantation development. Fertility and sterility.

[30] Grygoruk C (2011). Influence of embryo transfer on blastocyst viability. Fertility and sterility.

[31] Grygoruk C (2011). Pressure changes during embryo transfer. Fertility and sterility.

[32] Bulletti C (2000). Uterine contractility during the menstrual cycle. Human reproduction.

